# The effects of living environment on disaster workers: a one–year longitudinal study

**DOI:** 10.1186/s12888-016-1058-4

**Published:** 2016-10-21

**Authors:** Masanori Nagamine, Nahoko Harada, Jun Shigemura, Kosuke Dobashi, Makiko Yoshiga, Naoki Esaki, Miyuki Tanaka, Masaaki Tanichi, Aihide Yoshino, Kunio Shimizu

**Affiliations:** 1 Division of Behavioral Science, National Defense Medical College Research Institute, Saitama, Japan; 2 Department of Nursing Science of Community Health Care System, Tohoku University School of Health Sciences, Miyagi, Japan; 3 Department of Psychiatry, National Defense Medical College, Saitama, Japan; 4 Camp Kokura, Japan Ground Self-Defense Force, Fukuoka, Japan; 5 Camp Beppu, Japan Ground Self-Defense Force, Oita, Japan; 6 Camp Kurume, Japan Ground Self-Defense Force, Fukuoka, Japan; 7 Camp Omura, Japan Ground Self-Defense Force, Nagasaki, Japan

**Keywords:** Disaster workers, Great East Japan Earthquake, Post-traumatic stress response, Psychological distress, Risk factors, Living environment

## Abstract

**Background:**

Defense Force workers engaged in disaster relief activities might suffer from strong psychological stress due to the tasks that they had been involved. We evaluated how living environments, work environments, and individual factors psychologically affect those who engaged in disaster relief activities.

**Method:**

Data generated with 1506 personnel engaged in the Great East Japan Earthquake relief activity were analyzed. Those who scored ≥25 points on the Impact of Events Scale-Revised and the Kessler Psychological Distress Scale (K10) were allocated into the high post-traumatic stress response (high-PTSR) group, and the high general psychological distress (high-GPD) group, respectively.

**Results:**

The multiple logistic regression analysis extracted living environment (camping within the shelter sites) as the significant risk factor for both high-PTSR (OR = 3.39, 95 % CI 2.04–5.64, *p* < 0.001) and high-GPD (OR = 3.35, 95 % CI 1.77–6.34, *p* < 0.001) groups.

**Conclusion:**

It is desirable for disaster workers to have a living environment in which they can keep an appropriate distance from the victims.

## Background

Psychological impacts on disaster workers have been getting a lot more attention in the past decades [1]. Disaster workers are potentially exposed to severe work environments, inhumane situations, and overwhelming emotional reactions from victims. The concepts of secondary traumatic stress, compassion fatigue [2], and vicarious traumatization [3] have been used to describe psychological burdens in social workers. These psychological impacts can trigger various reactions, including depression and anxiety disorder, in addition to stress-related disorders, such as acute and post-traumatic stress disorder (PTSD) [4]. A recent meta-analysis on disaster relief workers reported that 10 % of the workers experience PTSD [5], indicating the seriousness of a psychologically traumatic experience. Young age, being single, previous experiences with natural disasters, poor social support, low educational level, and non-affiliated volunteer status are identified demographic risk factors [4–10]. On the other hand, maintaining professional distance with victims [11], and avoiding excessive empathy and identification [12–14], are reported as mitigating factors. Therefore, the living environment of disaster workers, such as staying on the same premises with the disaster victims, might increase the risk of psychological impacts; however, no study has yet investigated these issues. From the point of view of health management in the disaster, empirical research into such issues is urgently needed. This study is the first to evaluate how living and work environments, as well as individual factors, psychologically affect disaster relief workers.

## Method

### The Great East Japan earthquake

On 11 Mar 2011, Tohoku district of Japan was struck by a 9.0 magnitude earthquake. This triggered three-fold disasters consisting of earthquakes, tsunamis, and radiation leakage, which resulted in at least 18,000 deaths and missing persons [15]. Immediately after the earthquake occurred, the Japanese government decided to send more than 100,000 Self-Defense Force members to the stricken areas [16].

### Study participants

Approximately 2400 Japan Ground Self-Defense Force (JGSDF) members from the Northern Kyushu region, over 1500 km away from the afflicted areas, were dispatched to Kesennuma City and Minamisanriku Town in Miyagi Prefecture, which were severely affected by the tsunami. Most of the members departed from their home garrisons on 12 March, and engaged in search and rescue for missing persons until 14 May. After returning to their home garrisons, the mental health status of the troop members were evaluated at 1, 6, and 12 months post-mission completion. We excluded the data of those who could not complete this questionnaire because of relocation or other mission and those who had more than three missing demographic variables. The responses of 1506 members were used in the final analysis.

### Living and working environment of disaster workers

The troops were based in two different types of camps: camps remotely located from the affected area (JGSDF facility for field exercise; approximately 80 km away from the working area) and camps located in the affected area such as the track fields of schools that were converted to shelters for disaster victims. Since there were insufficient places in the latter camps, some troop members shared their premises with the disaster victims. Due to organizational strict discipline, the members were ordered to refrain from smoking cigarettes and avoid casual conversations in the camps because of their exposure to the public eye.

The troops engaged in search and rescue mission experienced frequent aftershocks. During their activity, there were 659 aftershocks with magnitudes greater than 5.0 [17], and the daily mean temperature ranged from −1.4 to 15.9 °C [18]. Moreover, almost all facilities of fishery and marine product processing, the main industry of the coastal regions, were absolutely destroyed; rotten fish and its product diffused foul smells that produced non-life threatening but disturbing environments for people [19]. Under such harsh conditions, JGSDF members discovered 521 bodies in this region; most of whom drowned. These included children’s bodies and very badly damaged bodies.

### Psychological evaluation

The survey was conducted by JGSDF clinical psychologists at 1, 6, and 12 months post-mission completion. The Japanese versions of the Impact of Events Scale-Revised (IES-R) [20] and the Kessler Psychological Distress Scale (K10) [21] were used for the psychological evaluation scales. The IES-R is a 22-item self-administered questionnaire used to evaluate post-traumatic stress response (PTSR). It comprises three major PTSD symptoms as subscales: intrusion, avoidance, and hyperarousal [22]. According to a previous report [20], those scoring 25 points or higher were referred to as the high-PTSR group.

The K10 is a ten-item self-administered questionnaire developed by Kessler [23]. It is based on the Item Response Theory and is widely used as a tool for evaluating general psychological distress (GPD) [24, 25]. This measure uses a 24/25-point cutoff value as a screening test for psychological illnesses [26]. As such, those scoring 25 points or higher were considered to be the high-GPD group.

In addition to the two psychological measurements, their living environment factors (camping with only troop members or camping in an environment with disaster victims), working environment factors (dispatch period and whether or not they were exposed to corpses), and sociodemographic factors (age, gender, and rank) were investigated as independent variables.

### Statistical analyses

Regarding the collected data on IES-R and K10, no data showed normality or equal variance; therefore, non-parametric tests were conducted for the chronological analyses (Friedman test and Cochran’s Q test) and univariate analyses (Kruskal-Wallis test for age and Mann–Whitney *U* test for others) on these measures. Bonferroni correction was used for multiple comparisons. Finally, multiple logistic regression analyses were conducted to investigate factors related to the high-risk groups measured by IES-R and K10. In the analyses, those who scored ≥25 on IES-R and K10 at least once during the research period were allocated into high-PTSR group (*N* = 71) and high-GPD group (*N* = 43), respectively. Since we required at least 10 events of these outcome measures per variable to avoid an overfit model on logistic regression analyses [27], factors that showed at least one or more *p* value of <0.2 in the univariate analysis were designated as independent variables. We used the forced entry method and the significance level for the tests was set as *p* < 0.05. IBM SPSS Statistics version 22 was used for the statistical tests.

## Results

### Participants’ characteristics

Table 1 shows the participants’ characteristics. About two-thirds (67.8 %) of the participants were dispatched for 1 to 3 months, with the rest being dispatched for less than 1 month. Approximately 70 % of the troop members were exposed to corpses. While most of the troop members were living in an environment only with other members, some of the members (16.8 %) were sharing the same premises with the disaster victims.

**Table 1:** Participants’ characteristics and the results of univariate analyses for each attribute

			IES-R									K10								
			1 month later			6 months later			12 months later			1 month later			6 months later			12 months later		
	*N*	%	Mean	s.d.	*p* value	Mean	s.d.	*p* value	Mean	s.d.	*p* value	Mean	s.d.	*p* value	Mean	s.d.	*p* value	Mean	s.d.	*p* value
Age																				
< 30	711	47.2	6.6	8.3	0.42	3.3	4.9	0.77	2.8	4.4	0.23	13.0	4.7	0.24	11.1	2.7	0.43	11.0	2.4	0.55
30 to 39	311	20.7	5.8	7.0		2.9	4.6		2.8	4.5		12.5	3.4		10.7	2.0		11.0	2.2	
≥ 40	484	32.1	5.9	7.3		3.1	4.4		3.2	5.1		12.3	3.7		11.1	2.4		11.2	2.5	
Gender																				
Male	1485	98.6	6.2	7.7	0.18	3.1	4.7	0.72	2.9	4.7	0.14	12.6	4.2	0.81	11.0	2.5	0.45	11.1	2.4	0.56
Female	21	1.4	4.3	6.7		3.7	5.0		3.3	3.5		12.6	4.0		11.0	3.1		11.1	1.9	
Rank																				
Officer	75	5.0	5.3	6.5	0.43	4.2	5.9	0.15	3.5	5.6	0.30	13.3	5.0	0.28	11.6	2.9	0.09	11.3	2.4	0.10
Private/Sergeant	1431	95.0	6.3	7.8		3.1	4.6		2.9	4.6		12.6	4.1		11.0	2.5		11.0	2.4	
Dispatch period																				
< 1 month	485	32.2	5.8	7.6	<0.05	2.8	4.3	<0.05	2.9	4.6	0.64	12.4	4.0	<0.05	10.8	2.2	<0.01	11.1	2.2	<0.05
1 to 3 months	1021	67.8	6.4	7.8		3.3	4.8		3.0	4.7		12.8	4.2		11.1	2.6		11.0	2.5	
Exposure to corpses																				
Present	1030	68.4	6.8	7.9	<0.001	3.4	4.8	<0.001	3.0	4.8	0.19	12.7	4.3	0.41	11.1	2.6	0.41	11.0	2.3	0.07
Not present	476	31.6	5.0	7.1		2.6	4.3		2.8	4.4		12.5	3.9		11.0	2.3		11.2	2.6	
Living environment																				
Only with troop members	1253	83.2	5.7	7.3	<0.001	2.9	4.2	<0.01	2.7	4.4	<0.001	12.4	3.9	<0.001	10.9	2.1	<0.001	11.0	2.2	0.08
Sharing the premises with the victims	253	16.8	8.9	9.3		4.2	6.5		4.1	5.8		13.8	5.3		11.9	3.8		11.4	3.1	

*IES-R* Impact of Event Scale-Revised, *K10* Kessler Psychological Distress Scale

Statictics: Kruskal-Wallis test for age and Mann–Whitney *U* test for others

### Chronological data analyses on IES-R and K10

The chronological data of mean IES-R and K10 scores for all participants is shown in Table 2. A significant correlation was seen between the IES-R and K10 scores for all measurement periods (Spearman’s rank correlation test; 1 month: *r* = 0.614, *p* < 0.001, 6 months: *r* = 0.484, *p* < 0.001, 12 months: *r* = 0.456, *p* < 0.001). All the IES-R related scores (total, intrusion, avoidance, hyperarousal, ratio of high-PTSR group) at 6 and 12 months significantly decreased from the score at 1 month (Friedman and Cochran’s Q test, multiple comparisons with Bonferroni correction: 1 month > 6 months, 12 months, *p* < 0.001).

**Table 2:** Chronological data analyses on IES-R and K10

	1 month	6 months	12 months	*P* value
IES-R total, mean (s.d.)	6.2 (7.7)	3.1 (4.7)	2.9 (4.7)	<0.001^c^
IES-R intrusion, mean (s.d.)	2.4 (3.1)	1.3 (2.0)	1.2 (1.9)	<0.001^c^
IES-R avoidance,mean (s.d.)	2.3 (3.6)	1.1 (2.3)	1.0 (2.2)	<0.001^c^
IES-R hyperarousal, mean (s.d.)	1.5 (2.3)	0.7 (1.5)	0.7 (1.4)	<0.001^c^
High-PTSR^a^ group, *n* (%)	56 (3.7)	12 (0.8)	11 (0.7)	<0.001^d^
K10, mean (s.d.)	12.6 (4.2)	11.0 (2.5)	11.1 (2.4)	<0.001^c^
High-GPD^b^ group, *n* (%)	33 (2.2)	8 (0.5)	7 (0.5)	<0.001^d^

*IES-R* Impact of Event Scale-Revised, *PTSR* post-traumatic stress response, *K10* Kessler Psychological Distress Scale, *GPD* general psychological distress

^a^Defined according to the Japanese version of the Impact of Events Scale-Revised (≥25)

^b^Defined according to the Japanese version of the Kessler Psychological Distress Scale (≥25)

^c^Friedman test, Multiple comparisons with Bonferroni correction: 1 month > 6 months, 12 months, *p* < 0.001

^d^Cochran’s Q test, Multiple comparisons with Bonferroni correction: 1 month > 6 months, 12 months, *p* < 0.001

Similar to IES-R, K10 scores and ratio of high-GPD group significantly decreased from 1 to 6 months and 12 months post-mission (Friedman and Cochran’s Q test, multiple comparisons with Bonferroni correction: 1 month > 6 months, 12 months, *p* < 0.001).

### Univariate analyses

The results of the univariate analyses for each participant’s attributes are also shown in Table 1. No significant correlations were found regarding age, gender, or rank for either IES-R or K10. Concerning the dispatch period, a significant difference was found in most of the psychological measures except for the IES-R conducted at 12 months. The group that experienced longer dispatch periods had a significantly high score. The group with exposure to corpses had significantly higher IES-R scores at 1 and 6 months compared to the group without exposure. Concerning living environment, significant differences were seen for most of the psychological measures except the K10 score at 12 months. The group that shared the same premises with the disaster victims had significantly higher scores than the group that lived with only dispatched troop members.

### Multiple logistic regression analyses

Table 3 shows the multiple logistic regression analysis results. In the univariate analysis, most of the independent variables showed at least one or more *p* value of <0.2 except for age on both IES-R and K10, and gender on K10. Therefore, age was excluded as an independent variable in the multiple logistic regression analysis for High-PTSR and age and gender in the analysis for High-GPD.

**Table 3:** Results of multiple logistic regression analyses

		Reference	β	s.e.	Adjusted OR	95 % CI	*P* value
High-PTSR^a^							
Gender	Female	(Male)	0.13	1.05	1.13	0.15–8.83	0.90
Rank	Officer	(Private/Sergeant)	0.30	0.54	1.35	0.47–3.86	0.57
Dispatch period	1 to 3 months	(<1 month)	−0.32	0.27	0.73	0.43–1.22	0.23
Exposure to corpses	Present	(Not present)	0.21	0.28	1.23	0.71–2.15	0.47
Living environment	Sharing the premises with the victims	(Only with troop members)	1.22	0.26	3.39	2.04–5.64	<0.001
High-GPD^b^							
Rank	Officer	(Private/Sergeant)	0.53	0.62	1.69	0.50–5.69	0.40
Dispatch period	1 to 3 months	(<1 month)	0.33	0.38	1.39	0.66–2.90	0.38
Exposure to corpses	Present	(Not present)	0.00	0.36	1.00	0.50–2.00	0.99
Living environment	Sharing the premises with the victims	(Only with troop members)	1.21	0.33	3.35	1.77–6.34	<0.001

*PTSR* post-traumatic stress response, *GPD* general psychological distress, *OR* odds ratio

^a^Defined as those who scored ≥25 on the Japanese version of the Impact of Events Scale-Revised at least once during the research period (*N* = 71)

^b^Defined as those who scored ≥25 on the Japanese version of the Kessler Psychological Distress Scale at least once during the research period (*N* = 43)

For both the high-PTSR and GPD groups, living environment was extracted as the significant risk factor. The group sharing premises with the disaster victims was 3.39 times more likely to be in the high-PTSR and 3.35 times in the high-GPD groups than those who camped with only troop members.

## Discussion

We examined the psychological effects of living environment factors, working environment factors, and individual factors on JGSDF troop members who engaged in disaster relief activities following the 2011 Great East Japan Earthquake. Dispatched troop members who shared the same premises as the disaster victims were 3.4 times more likely to be in the high-PTSR and GPD groups compared to those who lived with only dispatched troop members. Tasks that involved the handling of corpses [7, 8, 12–14, 28–30] and long-term relief activities [9, 31] have been reported to have large psychological impacts, but this study did not indicate the same results of previous studies. These results imply that not direct but indirect traumatic exposure through relationships with disaster victims could have greater influence on the mental health of disaster workers.

### The effects of living environment on disaster workers

Our results suggest that living environment was the most significant risk factor to participants’ post-disaster mental health in particular in this study. To our knowledge, no other study has uncovered such a result. Disaster relief workers potentially experience secondary trauma through their activities. Their outcomes have been reported in many studies in relation to PTSD [5, 32]. Ursano and colleagues have shown the importance of “identification with disaster victims” as a mechanism by which relief workers experience secondary trauma [14]. Cetin and others also reported results that support this finding [12] and warned against excessive identification with disaster victims.

Work-related trauma exposure has been mentioned in various professions. A study on emergency medical personnel reported the importance of having sympathy while maintaining emotional distance as a coping mechanism [33]. In a review of caregivers of torture victims, Pross discussed the importance of keeping a balance between an appropriate professional distance and empathy [11]. In our study, the troop members who lived on the same premises with the disaster victims had many chances of coming into contact with them even after they returned from work. This suggests that they were at a higher risk of experiencing excessive empathy and identification, and had great difficulty keeping their emotional distance from the victims. As a result, living environment may be the largest risk factor for both high-PTSR and GPD.

There are other possible effects of sharing living quarters with victims. In order to maintain the psychological health of disaster workers, the importance of self-care, including obtaining sufficient rest, engaging in non-work activities such as sports, and prioritizing relaxation has been advocated [11, 34]. However, in an environment where they are under the gaze of the victims, the relief workers were probably unable to relax enough even when their work was done for the day. Disaster workers under such conditions might have difficulties in relieving their stress.

### Possible factors that reduced psychological effects on disaster workers

Our results showed that less than 1 % of the participants were in the high-PTSR/GPD groups at 12 months, despite the devastating magnitude of the disaster and exposure to corpses. Meta-analytic studies on past disaster relief activities reported that the PTSD prevalence rate was approximately 10 % [5]. Although our study is not directly comparable to these studies, our results show extremely little lasting psychological impact. We propose three possible explanations for this trend.

Firstly, JGSDF members received high praise and much media appreciation for their efforts in the Great East Japan Earthquake [35], and this experience might have mitigated the negative psychological impacts of the experience [16]. In a study of firefighters who responded to the 1995 Great Hanshin Earthquakes, Kato and colleagues stated that “giving sufficient social praise and support toward disaster workers’ activities is extremely significant in reducing the psychological impacts” [36].

Second, this study’s participants were troop members deployed outside of the affected areas and were not residing in the disaster areas. Past reports have shown that those dispatched to provide support from within the afflicted areas had significantly more traumatic reactions compared to those dispatched from outside the afflicted areas [36].

Third, a stronger motivation for the personnel’s mission might be associated with their lower psychological responses. The Great East Japan Earthquake caused unprecedented damage to Japan, and resulted in the largest scale disaster dispatch ever for the JGSDF. Strong morale and a sense of meaningful mission were implied in many statements made by several troop members who engaged in these activities, such as, “I am a troop member for occasions like this” [37]. Feelings of strong morale and a sense of meaningful mission can increase resilience and maintain mental health among relief workers [38, 39]. These factors may have reduced the emotional impacts felt by the troops, although we were unable to objectively evaluate this hypothesis. Future studies are needed to conduct a quantitative analysis of the effects of strong morale and a sense of mission.

### Limitations

Our longitudinal study has several limitations. First, although our results suggest the importance of living environment on disaster workers, we investigated whether they have stayed only with troop members or have been sharing the premises with the victims. Therefore, detailed off-hours exposures to traumatic event (e.g. to what extent they have contact disaster victims or witnessed their hardship) were neither assessed nor quantified. Similarly, we evaluated whether or not they have exposed to dead bodies and have not quantified traumatic exposures during their on-duty task assignments (e.g. numbers of bodies, or numbers of hours of exposure to bodies).

Second, this study does not represent all troops engaged in Great East Japan Earthquake or other disaster relief activities. The participants’ psychological impacts were much less severe compared to those in studies of other disasters. This trend can be attributed to various reasons. This study employs a registered questionnaire, which has been reported to influence participants to conceal their symptoms, as compared to an anonymous questionnaire [40]. Furthermore, the Japanese sociocultural background strongly stigmatizes the expression of emotional suffering [41]. Thus, there is a great possibility that the troop members’ psychological impacts are underreported in this study.

Finally, this study did not examine other stress-related factors, such as marital status, medical history, or major life events. Further study including such information is needed to obtain more rigorous mental health effects of these factors on disaster workers.

## Conclusion

Our findings demonstrated that the living environment of disaster relief workers is associated with their adverse mental health. This result has significant safety implications for planners of disaster relief activities. Past reports on disaster relief activities mainly focused on work details and individual factors as the risk factors with the most psychological impact. Our results, in addition to this, suggest the importance of separating disaster relief workers’ living environment from that of the victims. This process might be helpful for maintaining an emotional distance from disaster victims, and to avoid excessive empathy and identification with victims. Independence from these environment would facilitate workers to perform sufficient self-care, which is potentially effective for the prevention of adverse mental health.

Situations that increase the risk of secondary trauma are not limited to disaster relief activities but also humanitarian aid activities, including medical and welfare activities. Further studies concerning secondary trauma need to be accumulated in order to safely manage the mental health of the people who engage in such activities.

## Abbreviations

GPD: General psychological distress
IES-R: Impact of Events Scale-Revised
JGSDF: Japan Ground Self-Defense Force
K10: Kessler Psychological Distress Scale
PTSD: Post-traumatic stress disorder
PTSR: Post-traumatic stress response
